# Venous thromboembolism and intracranial hemorrhage in patients undergoing glioblastoma surgery

**DOI:** 10.1038/s41598-023-48542-2

**Published:** 2023-12-07

**Authors:** Florian Wilhelmy, Michael Gaier, Uwe Planitzer, Johannes Kasper, Gordian Prasse, Clara Frydrychowicz, René Oesemann, Jürgen Meixensberger, Dirk Lindner

**Affiliations:** 1 Department of Neurosurgery, University Hospital Leipzig, Liebigstrasse 20, 04103 Leipzig, Germany; 2 Department of Anesthesiology and Intensive Care, University Hospital Leipzig, Liebigstrasse 20, 04103 Leipzig, Germany; 3 Division of Neuropathology, University Hospital Leipzig, Liebigstrasse 26, 04103 Leipzig, Germany; 4 Division of Neuroradiology, University Hospital Leipzig, Liebigstrasse 20, 04103 Leipzig, Germany

**Keywords:** Surgical oncology, CNS cancer

## Abstract

In the perioperative management of patients with glioblastoma (GBM), physicians face the question of whether and when to administer prophylactic or therapeutic anticoagulation (AC). In this study, we investigate the effects of the timing of postoperative heparinization on thromboembolic events (TE) and postoperative hemorrhage (bleeding, PH) as well as the interactions between the two in the context of an underlying intracerebral malignancy. For this retrospective data analysis, 222 patients who underwent surgery for grade IV glioblastoma, IDH-wildtype (2016 CNS WHO) between 01/01/2014 and 31/12/2019 were included. We followed up for 12 months. We assessed various biographical and clinical data for risk factors and focused on the connection between timepoint of AC and adverse events. Subgroup analyses were performed for pulmonary artery embolism (PE), deep vein thrombosis, and postoperative intracranial hemorrhage (PH) that either required surgical intervention or was controlled radiologically only. Statistical analysis was performed using Mann–Whitney U-Test, Chi-square test, Fisher’s exact test and univariate binomial logistic regression. *p* values below 0.05 were considered statistically significant. There was no significant association between prophylactic AC within 24 h and more frequent major bleeding (*p* = 0.350). AC in patients who developed major bleeding was regularly postponed by the physician/surgeon upon detection of the re-bleeding; therefore, patients with PH were anticoagulated significantly later (*p* = 0.034). The timing of anticoagulant administration did not differ significantly between patients who experienced a thromboembolic event and those who did not (*p* = 0.634). There was considerable overlap between the groups. Three of the six patients (50%) with PE had to be lysed or therapeutically anticoagulated and thereafter developed major bleeding (*p* < 0.001). Patients who experienced TE were more likely to die during hospitalization than those with major bleeding (*p* = 0.022 vs. *p* = 1.00). Prophylactic AC within 24 h after surgery does not result in more frequent bleeding. Our data suggests that postoperative intracranial hemorrhage is not caused by prophylactic AC but rather is a surgical complication or the result of antithrombotic therapy. However, thromboembolic events worsen patient outcomes far more than postoperative bleeding. The fact that bleeding may occur as a complication of life-saving lysis therapy in the setting of a thromboembolic event should be included in this cost–benefit consideration.

Neurosurgical patients are at high risk for both thromboembolic events and postoperative intracranial hemorrhage^1–4^. This applies especially for patients’ undergoing glioma surgery. As a practitioner, one is caught between a rock and a hard place when it comes to the onset of prophylactic anticoagulation, for both adverse events impair patients’ outcomes significantly^5–7^. Risk factors for both bleeding and thromboembolic events have been largely examined. To prevent thromboembolism, prophylactic anticoagulation with low molecular weight heparin and its derivatives is standard in perioperative care^6–8^. There are various studies on whether or not to apply chemoprophylaxis or mechanical devices such as intermittent pneumatic compression boots in patients with intracranial pathologies^9,10^. Yet, there is scarce data on *when* to apply said prophylaxis^11,12^.

The rare cases in which pulmonary artery embolism occurs are associated with high mortality^13^. Causal therapy by means of therapeutic anticoagulation or lysis therapy is crucial here, but is used only very hesitantly due to the risk of bleeding. Due to its relatively scarce occurrence, there is no evident data on the application of full-dose anticoagulation in these cases.

The main objective of this study is to investigate the impact of the timing of prophylactic anticoagulation on serious adverse events, thereby contributing to a better understanding of the need for and risk of postoperative anticoagulation. In addition, the interaction between these two adverse events will be further elucidated. Subsequently, suggestions for conclusive prospective research should be developed in order to define precise guidelines.

## Methods

The study was approved by the ethics committee of the Medical Faculty, University of Leipzig (No. 053/19-ek). The need for informed consent was waived by the ethics committee of the University of Leipzig. Apart from the patient selection, methodology has been described before^8,11,12^. I confirm that all methods were performed in accordance with the relevant guidelines and regulations.

### Patient selection

All patients who underwent surgery for grade IV glioblastoma, IDH-wildtype (according to the 2016 World Health Organization Classification of Tumors of the Central Nervous System) from 01/01/2014 to 31/12/2019 were identified using the digital patient database of the University Hospital of Leipzig. We started data analysis on 01/03/2019. All patient data were fully anonymized. Being an anonymous retrospective review with no personal data, informed consent was not required. Inclusion criteria were age above 18 years and conclusive documentation on the initiation of anticoagulation. 222 patients were included.

Diagnosis of intracranial bleeding and thromboembolism.

Magnetic resonance imaging (MRI) or alternatively 5 mm and 1.25 mm computed tomography (CT) scans were regularly performed within 24 h after surgery if contraindications to MRI were present. Patients displaying new neurological deficits or insufficient awakening after surgery were CT-scanned immediately.

Postoperative hemorrhage was defined as radiologically diagnosed intracranial residual blood. Re-Bleeding was defined independently by an experienced neuroradiologist and distinguished between residual blood and re-bleeding. This definition was reviewed again during data assessment for this study. We identified both major and minor bleedings to increase sensitivity for PH. Major bleeding was defined as bleeding that either required surgery or would have required surgery in a setting other than palliative. Minor bleeding was defined as bleeding that did not require surgery and was controlled radiologically only. Unless stated otherwise, the statistical evaluation focuses on major bleedings.

Thromboembolic events were defined as pulmonary artery embolism or deep vein thrombosis detected by either CT scan or duplex sonography. Diagnostic methods were only initiated if symptoms occurred.

In one case, pulmonary artery embolism was diagnosed on the basis of clinical and echocardiographic features (sudden onset of severe dyspnea, most severe right heart strain on echocardiography and the need for cardiopulmonary resuscitation). The patient died before any conclusive imaging was performed.

We also examined cases in which intracranial ischemia occurred. In all cases, the infarctions could be attributed to the access pathways or complications during resection. Therefore, they were not considered to be thromboembolic events.

### Anticoagulation regimen

All cases admitted to our neurooncological center were discussed in a weekly, interdisciplinary tumor board, where the therapy regimen regarding surgical treatment or conservative procedure was decided based on international guidelines [EANO]. Patients were anticoagulated in accordance with national guidelines^14,15^, known independent risk factors, and pre-existing diseases, as well as body weight. The timing and dose as well as the choice of substance were decided according to the hospital’s guidelines and in interdisciplinary bedside discourse with intensive care and neurosurgical practitioners. The fact that anticoagulation did not follow a definite protocol resulted in the variety of timings and therapy regimens examined in this study. However, the common course of treatment and diagnosis can be described as follows:

Patients are not anticoagulated until CT or MR imaging of the head is obtained, which is routinely done on the first postoperative day (or within 24 h after surgery). CT scans are performed immediately following the onset of new neurological deficits. We begin prophylactic AC treatment in patients undergoing intracranial surgery on the first day after surgery, as long as postoperative CT or MR imaging do not show any signs of residual bleeding. Patients with postoperative or perioperative hemorrhage are mainly anticoagulated after two to five days following a repeat CT scan without any sign of a growing hemorrhage. In patients with a preoperative regimen of direct oral anticoagulants (DOAC) or vitamin K antagonists (VKA), therapy was interrupted for up to three weeks depending on indications during the period concerned. In these cases, heparin bridging was regularly performed and paused the day prior to surgery. Since 2014, a standard operating procedure has stated that hospitalized tumor patients should be considered for anticoagulation as long as there are no contraindications, in which case either mechanical prophylaxis or no prophylaxis at all should be applied. We have adapted this regimen and administer prophylactic anticoagulation with low-molecular-weight or unfractionated heparin to all eligible inpatients until the day before surgery.

## Assessed data

### Baseline data

Gender; smoking; diabetes; hypertension; atrial fibrillation; previous resection; paresis of lower limbs before surgery (defined according to JANDA ≤ 4); therapeutic anticoagulation in patient medication; platelet aggregation inhibitor in patient medication; American Society of Anesthesiologists (ASA)—score before surgery; age; body-mass-index (BMI).

### Perioperative data

Necessity for blood products intraoperatively (fresh frozen plasma, platelet concentrates, or red cell concentrates); intraoperative positioning (abdominal vs back-positioning); extent of resection (biopsy vs. subtotal/grosstotal resection); usage of steroids at intensive care unit (ICU); administration of prophylactic anticoagulation before surgery; duration of surgery (measured in minutes); highest systolic blood pressure intraoperatively (measured in mmHg); lowest body temperature intraoperatively (measured in degree Celsius); intensive care unit (ICU) time after surgery (measured in hours).

### Anticoagulation regimen

Timing of preoperative heparinization (measured in hours); timing of postoperative heparinization (measured in hours).

### Outcome.

#### Postoperative intracranial hemmorrhage

Major bleeding: radiologically defined, requiring surgical revision; Minor bleeding: radiologically defined, not requiring surgical revision.

#### Thromboembolic events

Pulmonary artery embolism, deep vein thrombosis.

#### Death and functional status

Need for cardiopulmonary resuscitation (CPR); in-hospital mortality; mortality after 30 days, three, six and twelve months; Modified Rankin Scale (MRS) at discharge and after three months.

### Statistical analysis

To describe the cohort, continuous parameters are presented as median, mean and with standard deviation; nominal parameters are displayed as percentage.

The population was dichotomously branched by the occurrence or nonoccurrence of adverse events (intracranial hemorrhage and thromboembolic event).

Continuous and ordinal parameters were evaluated with Mann–Whitney U-Test. Dichotomous parameters were analyzed using Fisher’s exact test. *p* values lower than 0.05 were considered statistically significant. Odd’s Ratios and their 95% confidence interval were calculated with univariate binomial logistic regression.

All analyses were computed using IBM SPSS Statistics software version 24 (IBM, Armonk, New York State, USA).

## Results

### Baseline data

There was no statistically significant difference in baseline and epidemiological data between both groups (Table 1).

**Table 1:** Baseline data, risk factors and outcome in patients with and without PH (postoperative intracranial hemorrhage)

Characteristics^1^	ICH (N = 9)	No ICH (N = 213)	p-value^2^
Sex (female)	3 (33.3%)	77 (36.1%)	1.00
Age (years)	69.56 ± 9.46	65.44 ± 11.29	.264
BMI (kg/m^2^)	26.74 (± 4.64)	26.05 (± 3.88)	.448
Smoker	0	31 (14.6%)	.366
Diabetes	2 (22.2%)	26 (12.2%)	.317
Hypertension	6 (66.7%)	129 (60.6%)	1.00
Atrial fibrillation	0 (0.0%)	16 (7.5%)	1.00
Previous resection	2 (22.2%)	53 (24.9%)	1.00
Therapeutic AC in patient medication	0 (0.0%)	13 (6.1%)	1.00
Anti-platelet-therapy in patient medication	1 (11.1%)	34 (16.0%)	1.00
American Society of Anesthesiologists Score < 3	5 (55.6%)	137 (64.3%)	.726
Preoperative prophylactic AC with LMWH	8 (88.9%)	181 (85.8%)	.676
Preoperative prophylactic AC with LMWH (Hours)	17.63 (± 8.31)	19.97 (± 9.40)	.676
Extent of resection (biopsy)	1 (11.1%)	22 (10.3%)	1.00
Duration of surgery (minutes)	331.56 (± 145.43)	224.15 (± 92.39)	0.28*
Lowest body temperature intraoperatively (°C)	35.68 (± .34)	36.09 (± .47)	.005*
Blood products intraoperatively	2 (22.2%)	1 (0.3%)	.004*
Highest sys. blood pressure intraoperatively (mmHg)	142.78 (± 29.17)	131.95 (± 15.83)	.331
Initiation of prophylactic AC after surgery (hours)	32.62 (± 14.18)	50.67 (± 30.25)	.034*
Time spend at intensive care unit (hours)	237.38 (± 183.57)	42.43 (± 69.08)	< .001***
Thromboembolic event	3 (33.3%)	5 (2.3%)	.002**
Therapeutic AC within 24 h after surgery	0 (0.0%)	37 (17.7%)	1.00
Cardiopulmonary resuscitation	1 (11.1%)	4 (1.9%)	.188
In-hospital mortality	0 (0.0%)	7 (3.3%)	1.00
Death within 30 days	1 (11.1%)	13 (6.1%)	.419
Death within three months	2 (22.2%)	40 (18.7%)	.632
Modified Rankin Scale at discharge^3^	4	2	.208*
Modified Rankin Scale at three months < 4^3^	4	3	.273

*BMI* body mass index, *LMWH* low molecular weight heparine, *AC* anticoagulation

^1^Categorical variables are presented with the number and percentage of patients to whom the characteristic applies, Continuous variables are presented with the mean ± the standard deviation. ^2^Categorical variables are calculated using Fisher's exact test (2-sided); Continuous variables are calculated using Mann–Whitney U test (2-sided. ^3^Median values.

### Perioperative data

We found no significant difference in the distribution of maximum intraoperative systolic blood pressure and the incidence of major bleeding. Duration of surgery was significantly longer (U = 542.500, Z = -2.176, *p* = 0.028; OR = 1.009, 95% CI [1.003/1.014]) and intraoperative body temperature decreased in the hemorrhage group (U = 439.000, Z = − 2.732, *p* = 0.005; OR = 0.181, 95% CI [0.046 /0.711]).

### Anticoagulation regimen

Patients who experienced major bleeding were anticoagulated significantly later (U = 561.500, Z = − 2.109, *p* = 0.034; OR = 1.052, 95% CI [1.018/1.086]).

### Outcome

The occurrence of TE was correlated to PHrs (*p* = 0.002; OR = 20.800, 95% CI [4.013/107.813]) as was the intraoperative administration of blood products (*p* = 0.004; OR = 60.571; 95% CI [4.894/749.639]). While major bleeding was not more likely to lead to in-hospital death (*p* = 1.000; OR = 0.958; 95% CI [0.932/0.985]) and did not worsen functionality at discharge (*p* = 0.208; OR = 1.002; 95% CI [0.512/1.964]) or three months thereafter (*p* = 0.273, OR = 1.219; 95% CI [0.735/2.021]), it did result in longer ICU treatment (U = 143.500, Z = − 3.985, *p* < 0.001; OR = 1.009; 95% CI [1.004/1.014]) (Table 1).

### Individual time course

Three patients developed major bleeding after life-saving lysis therapy or therapeutic AC due to pulmonary artery embolism and thereby circulatory deterioration. Only one patient (11,8%) suffered major bleeding after administration of prophylactic AC. This case is nonetheless exceptional, as intracranial hemorrhage occurred 18 days after first LMWH application. All other cases had not been given any kind of anticoagulation when hemorrhage occurred (Table 2).

**Table 2:** Timing of anticoagulation and development of postoperative intracranial hemorrhage (PH).

Time point of PH	Timing of postoperative prophylactic AC with LMWH (hours)	TE prior to PH	Time point of postoperative lysis therapy (hours)	Time point of postoperative therapeutic AC (hours)	Location of PH
1	100				Residual tumor
3	72				Resection site
6		PE	.1		Resection site
6	50				Resection site
12	79				Resection site/SDH
24	48				Resection site
24	74				Resection site
42	26				Resection site
48		PE		24	Resection site/SDH
96		PE		48	Subgaleal/EDH
96	32				Residual tumor
117	32				Resection site
528	32				

*PH* postoperative hemorrhage, *AC* anticoagulation, *LMWH* low molecular weight heparine, *TE* thromboembolic event.

### Baseline data

Apart from BMI, which was higher in the thromboembolism group than in the comparison group (U = 403.000, Z = − 2.540, *p* = 0.009; OR = 1.182; 95% CI [1.022/1.366]), there was no other significant difference in epidemiologic and baseline data between both groups (Table 3).

**Table 3:** Baseline data, risk factors and outcome in patients with and without postoperative thromboembolic event (TE).

Characteristics^1^	VTE (N = 8)	No VTE (N = 214)	p-value^3^
Sex (female)	5 (62.5%)	77 (35.9%)	1.00
Age (years)	68.75 (± 9.90)	65.49 (± 11.28)	.452
BMI (kg/m^2^)	29.28 ± 3.15	25.96 (± 3.88)	.009**
Smoker	0	31 (14.5%)	.604
Diabetes	2 (25%)	26 (12.1%)	.266
Hypertension	6 (75%)	129 (60.3%)	.486
Atrial fibrillation	0	16 (7.5%)	1.00
Previous resection	0	55 (25.7%)	.205
Therapeutic AC in patient medication	0	13 (6.2%)	1.00
Anti-platelet-therapy in patient medication	2 (25%)	33 (15.4%)	.617
American Society of Anesthesiologists Score < 3	4 (50%)	138 (64.5%)	.463
Preoperative prophylactic AC with LMWH	6 (75%)	183 (86.3%)	.314
Preoperative prophylactic AC with LMWH (hours)	25 (± 14.11)	19.70 (± 9.16)	.419
Extent of resection (biopsy)	1 (12.5%)	22 (10.3%)	.589
Abdominal positioning intraoperatively	1 (12.5%)	22 (10.4%)	.593
Duration of surgery (minutes)	226.12 (± 93.40)	292.87 (± 163.11)	.183
Duration of surgery (PE, minutes)^2^	340.50 (± 153.63)	225.41 (± 93.60)	.036*
Initiation of prophylactic AC after surgery (hours)	33.39 (± 15.33)	32.50 (± 19.47)	.634
Time spend at Intensive Care Unit (hours)	46.65 (± 77.89)	127.25 (± 172.91)	.273
Lysis Therapy within 24 h after surgery	2 (25%)	0 (0%)	< .001***
Intracranial hemorrhage	3 (37.5%)	10 (3.1%)	.002**
Cardiopulmonary resuscitation	3 (37.5%)	2 (0.9%)	< .001***
In-hospital mortality	2 (25%)	5 (2.3%)	.022*
Death within 30 days	2 (25%)	12 (5.7%)	.087
Death within three months	2 (25%)	40 (20.1%)	.665
Modified Rankin Scale^3^ at discharge	4	3	.047*
Modified Rankin Scale^3^ at Three months	3.5	3	.110

*BMI* body mass index, *LMWH* low molecular weight heparine, *AC* anticoagulation

^1^Categorical variables are presented with the number and percentage of patients to whom the characteristic applies, Continuous variables are presented with the mean ± the standard deviation. ^2^Categorical variables are calculated using Fisher's exact test (2-sided); Continuous variables are calculated using Mann–Whitney U test (2-sided). ^3^Median values.

### Perioperative data

We found no significant difference in distribution of the operation time in the overall analysis (U = 610.500, Z = − 1.344, *p* = 0.183; OR = 1.006; 95% CI [1.000/1.012]). However, a significantly longer operative time was shown in the subgroup analysis of pulmonary artery emboli (U = 322.500; Z = − 2.078, *p* = 0.036; OR = 1.009; 95% CI [1.002/1.015]).

Anticoagulation regimen.

The timing of anticoagulation started postoperatively between the thromboembolism group and the comparison group did not differ significantly (U = 769.500, Z = − 0.486, *p* = 0.634; OR = 0.996, 95% CI [0.950/1.045]).

### Outcome

In-hospital mortality in the TE group was increased (*p* = 0.022; OR = 13.933; 95% CI [2.236/86.829]) as was the need for cardiopulmonary resuscitation (p =  < 0.001; OR = 63.600; 95% CI [8.634/468.491]). Functional capacity, which was measured by the modified Rankin scale, was lower in the TE group upon discharge from hospital (*p* = 0.047; OR = 1.929, 95% CI [1.126/3.299]) (Table 3).

### Individual time course

Four of the eight patients (50%) who developed TE were anticoagulated prior to it. None of the above events occurred within the first week after surgery. The remaining four events, all of which were pulmonary artery emboli, occurred within three days after surgery and were not previously anticoagulated (Table 4).

**Table 4:** Time course of anticoagulation and development of postoperativeTE (thromboembolic event) (TE).

Time point post-operative TE (hours)	Postoperative prophylactic AC with LMWH prior to TE (hours)	Therapeutic AC^1^	Lysis therapy^2^	Type of TE^3^	Cardiopulmonary resuscitation	Intracranial hemorrhage (hours)
1	no		Yes	PE^4^	Yes	6
24	no	UFH		PE		48
48	no	UFH		PE/DVT^5^		96
61	no		Yes	PE/DVT	Yes	no
264	51	UFH		PE/DVT		no
288	21		Yes	PE	Yes	no
336	26	Apixaban		DVT		no
504	28	LMWH		DVT		no

*AC* anticoagulation, *LMWH* low molecular weight heparine, *UFH* unfractioned heparine.

When grouped into AC initiation prior to or later than 48 h, we found no significant development of either TE (*p* = 0.149) or PH (*p* = 0.365) (Table 5). There was no significant difference in outcome when comparing time point of AC and mRS overall.

**Table 5:** Grouping into AC (anticoagulation) initiation before or later than 48 h showed no significant differences in developement of PH (postoperative hemorrhage) or TE (thromboembolic event).

Time point of AC	PH (n = 9)	No PH (N = 213)	p-value	TE (n = 8)	No TE (n = 224)	*p* value
< 48 h	7	184	0.365	5	183	0.149
> 48 h	2	29		3	39	

## Discussion

### Limitations

The study is limited by the rare occurrence of adverse events in general. A total of 17 adverse events occurred in 222 patients, some of which showed overlap. Under given circumstances, we found little significant evidence. Furthermore, the validity of the result decreases as the observation period progresses due to the high number of dropouts. This is certainly due in part to the severity of the underlying disease, which has a high mortality rate and often results in a palliative approach far from university hospital care. Another limiting factor are the large interindividual differences of preoperative functional status and previous diseases which continue to exist despite the narrowly defined patient clientele. Moreover, in the retrospective analysis, no conclusion can be drawn about the causality of the relationships.

### Early application of prophylactic anticoagulation has no impact on bleeding rates

We found no increase in occurrence of postoperative hemorrhage due to early application of prophylactic anticoagulation, which aligns with preliminary results^11,12,16–18^. Nonetheless, we found significant increase in major bleeding after administration of therapeutic AC or lysis therapy in vital indication (Table 2). The fact that prolonged duration of surgery and the need for intraoperative administration of blood products are related to an increased rate of bleeding events may seem trivial at first glance, as both may be consequences of intraoperative complications. However, these two factors may also be sensitizing for postoperative bleeding complications that occur later and thus play a warning role. Furthermore, this study supports the importance of perioperative temperature management, as hypothermia can lead to coagulopathy and thus increased bleeding^19^.

### Prophylactic anticoagulation as a mean to prevent thromboembolic events

We found no evidence for a positive impact of early prophylactic AC on TE rates. Unfortunately, or luckily, early postoperative adverse events in glioma patients are so rare, that bigger patients’ collectives are needed to detect statistical differences here. Furthermore, we have not compared groups receiving AC with patients not receiving any thromboembolic prophylaxis, as existing data clearly indicates the necessity of AC in perioperative setting^20–23^

### Adverse events and their impact on the overall outcome

The occurrence of TE as life-threatening events largely compromised patients’ immediate outcome (Table 3), whereas the occurrence of postoperative intracranial hemorrhage had no statistical impact on overall outcome (Table 1). As described before, we assume the occurrence of pulmonary embolism the more dreadful adverse event, compared to postoperative hemorrhage^11,12^. This has to be taken into account when defining AC regimen in elective glioma surgery.

## Conclusion

We conclude that early postoperative use (i.e. within 24 h after surgery) of prophylactic anticoagulation is safe in patients undergoing glioma surgery. We found no evidence underlining that pausing prophylactic AC is indicated. Regarding therapeutic anticoagulation or lysis therapy in life-threatening events under CPR conditions, hemorrhage seems to be a very regular complication. This especially applies to the early occurrence of PE (within 48 h after surgery). Regarding future research areas, inhibition of factor XI, which is currently in clinical development, represents a promising approach to prophylaxis of thromboembolic events while ensuring hemostasis^24,25^. In addition, there are interesting developments in catheter-based therapy of pulmonary artery embolism, which may become particularly relevant for neurosurgery^26^.

## Data Availability

The datasets used and/or analysed during the current study available from the corresponding author on reasonable request.
