# Author Correction: Venous thromboembolism and intracranial hemorrhage in patients undergoing glioblastoma surgery

**DOI:** 10.1038/s41598-024-60939-1

**Published:** 2024-05-06

**Authors:** Florian Wilhelmy, Michael Gaier, Uwe Planitzer, Johannes Kasper, Gordian Prasse, Clara Frydrychowicz, René Oesemann, Jürgen Meixensberger, Dirk Lindner

**Affiliations:** 1 Department of Neurosurgery, University Hospital Leipzig, Liebigstrasse 20, 04103 Leipzig, Germany; 2 Department of Anesthesiology and Intensive Care, University Hospital Leipzig, Liebigstrasse 20, 04103 Leipzig, Germany; 3 Division of Neuropathology, University Hospital Leipzig, Liebigstrasse 26, 04103 Leipzig, Germany; 4 Division of Neuroradiology, University Hospital Leipzig, Liebigstrasse 20, 04103 Leipzig, Germany

Correction to: *Scientific Reports* 10.1038/s41598-023-48542-2, published online 07 December 2023

The original version of this Article contained errors. An older version of the Table 1 and 3 was erroneously uploaded with an incorrect bigger subgroup of patients. The correct group size is 222 patients. As the result of this error, column ‘Total’ was removed from the tables.

The original Table 1 and 3 and accompanying legends appear below.

**Table 1:** Baseline data, risk factors and outcome in patients with and without PH (postoperative intracranial hemorrhage).

Characteristics^1^	Total (N = 331)	ICH (N = 13)	No ICH (N = 318)	*p* value^2^
Sex (female)	127 (38.4%)	4 (30.8%)	123 (38.7%)	0.773
Age (years)	64.29 ± 12.65	68.69 ± 9.13	64.11 ± 12.75	0.240
BMI (kg/m^2^)	26.20 ± 4	26.17 ± 4.03	26.20 ± 4.01	0.838
Smoker	50 (15.3%)	0	50 (15.9%)	0.231
Diabetes	45 (13.6%)	2 (15.4%)	43 (13.5%)	0.629
Hypertension	195 (58.9%)	9 (69.2%)	186 (58.5%)	0.570
Atrial fibrillation	23 (6.9%)	1 (7.7%)	22 (6.9%)	1.00
Previous resection	87 (26.3%)	2 (15.4%)	85 (26.7%)	0.526
Therapeutic AC in patient medication	22 (6.6%)	1 (7.7%)	21 (6.6%)	0.598
Anti-platelet-therapy in patient medication	43 (13.2%)	1 (8.3%)	42 (13.4%)	1.00
American Society of Anesthesiologists Score < 3	193 (59.2%)	8 (61.5%)	185 (59.1%)	1.00
Preoperative prophylactic AC with LMWH	244 (76.7%)	10 (76.9%)	234 (76.7%)	1.00
Preoperative prophylactic AC with LMWH (Hours)	22.66 ± 12.49	22.10 ± 11.95	22.68 ± 12.53	0.274
Extent of resection (biopsy)	45 (13.8%)	2 (16.7%)	43 (13.7%)	0.674
Duration of surgery (minutes)	226.94 ± 101.91	280.17 ± 159.45	224.92 ± 98.90	0.368
Lowest body temperature intraoperatively (°C)	36.05 ± .49	35.77 ± .44	36.07 ± .49	0.028*
Blood products intraoperatively	6 (1.8%)	2 (15.4%)	4 (1.3%)	0.020*
Highest sys. blood pressure intraoperatively (mmHg)	133.67 ± 18.23	144.62 ± 30.72	133.21 17.45	.295
Initiation of prophylactic AC after surgery (hours)	34.09 ± 15.02	54.50 ± 25.28	33.43 ± 14.16	< .001***
Time spend at intensive care unit (hours)	53.95 ± 86.85	206.15 ± 182.19	47.68 ± 74.79	< .001***
Thromboembolic event	8 (2.4%)	3 (23.1%)	5 (1.6%)	.002**
Therapeutic AC within seven days after surgery	13 (3.9%)	3 (23.1%)	10 (3.1%)	.011*
Cardiopulmonary resuscitation	5 (1.5%)	1 (7.7%)	4 (1.3%)	.183
In-hospital mortality	16 (4.8%)	1 (7.7%)	15 (4.7%)	.481
Death within 30 days	22 (7.2%)	2 (20%)	20 (6.8%)	.157
Death within three months	55 (19.5%)	3 (33.3%)	52 (19.0%)	.384
Modified Rankin Scale at discharge < 4	217 (65.8%)	4 (30.8%)	213 (67.2%)	.013*
Modified Rankin Scale at three months < 4	157 (60.6%)	3 (33.3%)	154 (61.6%)	.161

*BMI* body mass index, *LMWH* low molecular weight heparine, *AC* anticoagulation.

^1^Categorical variables are presented with the number and percentage of patients to whom the characteristic applies, Continuous variables are presented with the mean ± the standard deviation.

^2^Categorical variables are calculated using Fisher's exact test (2-sided); Continuous variables are calculated using Mann–Whitney U test (2-sided).

**Table 3:** Baseline data, risk factors and outcome in patients with and without postoperative thromboembolic event (TE).

Characteristics^1^	Total (N = 331)	VTE (N = 8)	No VTE (N = 323)	p-value^3^
Sex (female)	127 (38.4%)	3 (37.5%)	124 (38.4%)	1.00
Age (years)	64.29 ± 12.65	68.75 ± 9.90	64.18 ± 12.70	.351
BMI (kg/m^2^)	26.20 ± 4	29.28 ± 3.15	26.12 ± 4.00	.013*
Smoker	50 (15.3%)	0	50 (15.7%)	.613
Diabetes	45 (13.6%)	2 (25%)	43 (13.3%)	.298
Hypertension	195 (58.9%)	6 (75%)	189 (58.5%)	.479
Atrial fibrillation	23 (6.9%)	0	23 (7.1%)	1.00
Previous resection	87 (26.3%)	0	87 (26.9%)	.116
Leg paresis before surgery	64 (19.5%)	2 (25%)	62 (19.4%)	.656
Therapeutic AC in patient medication	22 (6.6%)	0	22 (6.8%)	1.00
Anti-platelet-therapy in patient medication	43 (13.2%)	2 (25%)	41 (12.9%)	.285
American Society of Anesthesiologists Score < 3	193 (59.2%)	4 (50%)	189 (59.4%)	.720
Preoperative prophylactic AC with LMWH	244 (76.7%)	6 (75%)	238 (76.8%)	1.00
Preoperative prophylactic AC with LMWH (hours)	22.66 ± 12.49	25.00 ± 14.11	22.60 ± 12.47	.726
Extent of resection (biopsy)	45 (13.8%)	1 (12.5%)	44 (13.8%)	1.00
Abdominal positioning intraoperatively	30 (9.1%)	1 (12.5%)	29 (9.1%)	1.00
Duration of surgery (minutes)	226.94 ± 101.91	292.88 ± 163.11	225.29 ± 99.75	.198
Duration of surgery (PE, minutes)^2^	226.94 ± 101.91	340.50 ± 153.63	224.82 ± 99.83	.042*
Initiation of prophylactic AC after surgery (hours)	34.09 ± 15.02	31.50 ± 13.33	34.12 ± 15.05	.494
Time spend at Intensive Care Unit (hours)	53.95 ± 86.85	127.25 ± 172.91	52.12 ± 83.30	.313
Therapeutic AC within seven days after surgery	10 (3%)	2 (25%)	8 (2.5%)	.021*
Lysis Therapy within seven days after surgery	2 (0.6%)	2 (25%)	0 (0%)	< .001***
Intracranial hemorrhage	13 (3.9%)	3 (37.5%)	10 (3.1%)	.002**
Cardiopulmonary resuscitation	5 (1.5%)	3 (37.5%)	2 (0.6%)	< .001***
In-hospital mortality	16 (4.8%)	2 (25%)	14 (4.3%)	.052
In-hospital mortality (PE)^2^	16 (4.8%)	2 (33.3%)	14 (4.3%)	.029*
Death within 30 days	22 (7.2%)	2 (25%)	20 (6.7%)	.107
Death within 30 days (PE)^2^	16 (4.8%)	2 (33.3%)	14 (4.3%)	.029*
Death within three months	55 (19.5%)	2 (25%)	53 (19.3%)	.656
Modified Rankin Scale at discharge < 4	217 (65.8%)	3 (37.5%)	214 (66.5%)	.128
Modified Rankin Scale at Three months < 4	157 (60.6%)	4 (50%)	153 (61%)	.716

*BMI* body mass index, *LMWH* low molecular weight heparine, *AC* anticoagulation.

^1^Categorical variables are presented with the number and percentage of patients to whom the characteristic applies, Continuous variables are presented with the mean ± the standard deviation.

^2^Categorical variables are calculated using Fisher's exact test (2-sided); Continuous variables are calculated using Mann–Whitney U test (2-sided).

The original Article has been corrected.

